# Stepping onto the stage: reflections of community-based participatory researchers using Forum Theatre to address the challenges of living with diabetes while experiencing homelessness

**DOI:** 10.3389/fpubh.2026.1744334

**Published:** 2026-03-20

**Authors:** Saania Tariq, Eshleen K. Grewal, Matt Larsen, Jeremy Auger, Roland Booth, Brian Bowdridge, Justin Lawson, Anna Whaley, Nathan G. Johnson, Tucker Reed, Hanan Bassyouni, David J. T. Campbell

**Affiliations:** 1 Department of Medicine, Cumming School of Medicine, University of Calgary, Calgary, AB, Canada; 2 Department of Community Health Sciences, Cumming School of Medicine, University of Calgary, Calgary, AB, Canada; 3 Calgary Diabetes Advocacy Committee, Calgary, AB, Canada; 4 Department of Cardiac Sciences, Cumming School of Medicine, University of Calgary, Calgary, AB, Canada

**Keywords:** diabetes, Forum Theatre, health, homelessness, participatory action research

## Abstract

The use of arts-based methods within health research is quickly growing in popularity. Forum Theatre is a unique type of performance that encourages audience participation to explore complex societal issues and is being used more frequently in participatory health research addressing social inequalities in health experiences and treatment. Using a Community-Based Participatory Research approach, The Calgary Diabetes Advocacy Committee (CDAC) drew on our collective experiences and used Forum Theatre to explore the challenges and possible solutions impacting people with lived experience of diabetes while living in an emergency housing shelter. Committee members participated in drama workshops and script development with rehearsals to produce and host two Forum events attended by 150 researchers, community members, and medical students. During these events, committee members performed a scripted dramatization of the challenges of managing diabetes in an emergency shelter after which attendees proposed potential interventions. A review of the field notes documented throughout three stages of our project, we identified reflections and challenges: (i) Drama workshops: Willingness to try something new; (ii) Script development and rehearsals: Tension in getting it right; and (iii) The Forum: Putting ourselves out there. Future health researchers considering Forum Theatre as an arts-based method are encouraged to draw on our experiences and reflections when planning and implementing their own productions.

## Introduction

A growing number of health researchers recognize that traditional written research outputs are often insufficient to meaningfully capture or communicate the nuances of emotions and the intricacies of peoples’ lived experiences (1). As a response to this identified limitation, some researchers are turning to the popular arts or arts-based methods (drama, video-making, photography, music, and dance). These media can result in long-lasting impressions through emotional or visceral responses among those engaging with the research, rather than solely cerebral or intellectual responses (2). One example of an arts-based method that was pioneered by educational and social work researchers and is increasingly being used in health research is a theatre-based research method called *Forum Theatre*.

The goal of Forum Theatre is to meaningfully engage communities and citizens to address power imbalances that lead to unjust treatment by creating a ‘Forum’. In this setting, the community members design and present an interactive performance where audience members are referred to as ‘spect-actors’. The production is engineered such that there is no traditional ‘fourth wall’ of popular theatre, which refers to an imaginary wall that delineates the audience as passive observers who are invisible to the actors on stage, and which thereby prevents the actors from interacting with the audience, and vice versa (3). The ‘fourth wall’ is removed by inviting spect-actors to actively intervene in a dramatized “act of oppression” that represents a shared story or lived experience of community members facing societal disadvantages and limited privilege (4). Typically, spect-actors first watch the play in its entirety. The play is then performed a second time, during which audience members are invited to stop the action, step into the scene, and propose or enact alternative responses aimed at addressing the issue being depicted. The interactive process to generate interventions enables audiences to engage with social or health-related issues from multiple perspectives, fostering critical reflection and dialogue around power dynamics, barriers to resources, and the lived experiences of marginalized or vulnerable groups.

In the context of health research, Forum Theatre can provide valuable insights into how individuals navigate healthcare systems, cope with illness, and challenge stigmatization, while also generating potential solutions as empowered audience members become co-creators in the research process (5). Previous research shows a range of applications for Forum Theatre in health contexts, from raising awareness and reducing stigma to fostering practical solutions and improving health and social services (6–9). Others have also used Forum Theatre to disseminate their research findings. For example, researchers from Minnesota, USA used Forum Theatre to showcase their findings from a Community-Based Participatory Research (CBPR) project developing behavioral interventions for people with type 2 diabetes and lived experience of homelessness (10). Across these applications, Forum Theatre is effectively adapted to address a variety of health issues and socio-cultural contexts. Specifically, Forum Theatre, a method which at its core addresses power imbalances and unjust treatment, is well situated to explore and address challenges with diabetes, given the incidence and prognosis of diabetes is strongly influenced by socioeconomic and sociocultural gradients (11–13). For example, diabetes incidence and long-term related health outcomes are associated with poverty, whereby those with lower income or unstable housing are more likely to develop diabetes and experience poorer related health outcomes, including emergency visits, heart disease, and death, compared to their housed, higher income counterparts (14, 15). As such, Forum Theatre is well suited to identify and address barriers to care for people with diabetes, especially those experiencing social disadvantages, such as homelessness.

Given the growing interest in arts-based research and the utility of Forum Theatre (5, 16, 17) the purpose of this commentary is to share our process and reflections of completing a Forum Theatre project to address the stigma faced by people with diabetes who experience homelessness. Since our goal is to share our process and reflections of our Forum Theatre project, the manuscript is organized using non-conventional headings, including context and methods, main text (which encompasses our process and reflections, including limitations and issues that arose), discussion, and conclusion.

## Context and methods

This work was guided by a CBPR approach with adults with diabetes who have experiences of homelessness in Calgary, Canada. CBPR was selected as the most appropriate approach as it centers leadership and expertise of people with lived expediencies, ensuring the research process and outcomes are grounded in community-identified needs and goals. Given the structural inequities and barriers faced by people experiencing homelessness, CBPR facilitates equitable collaboration and co-learning between academic researchers and people with lived experience, who may traditionally face poor representation and involvement in research. In our work, CBPR guided the creation and operations of the Calgary Diabetes Advocacy Committee (CDAC), a team comprised of co-researchers, or people with lived experience of diabetes and homelessness, research staff, students, and academic investigators. The formation of the group is detailed elsewhere (18). Briefly, in 2021, people with lived experience were recruited to the group by word of mouth and referrals from community organizations and partners. Once established, 25 focus groups, which consisted of semi-structured conversations with committee members who had diabetes and lived experience of homelessness, were held over the course of a year (2021–2022) to discuss barriers to diabetes management while experiencing homelessness (18). In addition to these conversations, we offered ‘Diabetes-101’ sessions held by the principal investigator, who is also a practicing Endocrinologist, and answered any question co-researchers had regarding diabetes pathophysiology and management. These sessions were offered to build capacity and provide a baseline understanding among committee members as we spoke about barriers to diabetes management. No other mental health or healthcare professionals were involved in the group activities as it was considered beyond the scope and purpose of our group. As outlined in our research ethics with the Conjoint Health Research Ethics Board at the University of Calgary (ID: REB20-0164), if concerns regarding co-researchers’ health and mental health came up, they were referred to appropriate community resources. All CDAC activities took place in private rooms that were booked at an inner-city public library and to respect the efforts and contribution of the co-researchers they received an hourly cash honorarium of $15–19 CAD for their participation, increasing $1-2/year as our grant-based funding allowed.

These focus group discussions informed our priority-setting activity in 2022, which identified “Diabetes Stigma and Awareness” as the collective priority to be addressed through a participatory action research project (19). After further discussion, the group chose to raise awareness through Forum Theatre and film that would focus on a person’s experience of managing their diabetes in a shelter. Details about the film are outlined elsewhere (20). Generally, our Forum Theatre project took place between September 2022 to June 2023 over three distinct stages, including drama workshops, script development and rehearsals, and the Forum. Drama workshops took place over a three-month period in 2022 where the facilitator guided the CDAC through multiple three-hour long drama workshops. Script development consisted of the committee holding three meetings to discuss specific “moments of oppression” to be incorporated into the script by a research assistant with a background in the performing arts (NGJ), who also directed and supported the Forum Theatre rehearsals. Lastly, we held two Forums. The first was in January 2023, in the same library room we used for the Forum Theatre workshops and rehearsals, with various community members and researchers taking on the role of spect-actors, and the second in June 2023 in a classroom where we had previously rehearsed, with undergraduate medical students taking on the role of spect-actors. Spect-actors during both Forums were given clear instructions on when and how to participate. Details about spect-actors’ interventions are described in the Main Text.

Throughout the drama workshops, script development and rehearsals, and the Forum, two research associates maintained descriptive, observational field notes documenting all activities. These field notes also included detailed minutes from meetings and debriefs among committee members, and sessions with the Forum Theatre Expert/Facilitator and the script writer/stage manager (NGJ). Reflexive field notes were additionally maintained throughout all stages of the Forum Theatre process by two research associates (ST and EG), the principal investigator (DC), and a community peer researcher (ML; a person with lived relevant lived experience and formal research training). Each contributor wrote field notes independently to avoid influencing one another’s reflections. Collected notes were mapped under three stages of our Forum Theatre experiences. Overarching summaries and themes that encapsulated the committee’s experiences were identified from the mapped data and are presented in the main text.

## Main text

Upon review of our field notes, we identified themes associated with various activities of pre-production and production of the Forum, including: (i) Drama workshops: Willingness to try something new; (ii) Script development and rehearsals: Tension in getting it right; and (iii) The Forum: Putting ourselves out there.

### Drama workshops: willingness to try something new

Starting a new activity was an exciting yet intimidating time for the committee, as the CDAC members did not have previous experience with the performing arts, and were generally uncertain if the chosen method of Forum Theatre would be an effective way to raise awareness about diabetes and homelessness. The purpose of these workshops was to strengthen our trust and communication with one another and build the skills needed for theatre: confidence, movement, improvisation, and memory. Committee members’ responses to the drama workshops were polarizing and depended upon the specific activities. On the one hand, individuals shared that they felt their physical, social, and emotional boundaries were being pushed by other group members and the facilitator during certain activities. On the other hand, however, many individuals expressed that they enjoyed the feeling of being out of their typical comfort zone during activities and they were generally happy to try something new.

Despite the facilitators’ encouragement to participate in the activities in ways which were comfortable, most people felt somewhat pressured to fully participate. Several expressed experiencing unwanted touch, pain (due to preexisting health conditions limiting their mobility or participation), and/or exacerbation of underlying anxiety as a result of their participation. For example, one committee member who had chronic back pain struggled to keep up with the group when the facilitator encouraged us to participate in relay races. When these situations arose, the facilitator allowed and encouraged modifications of the activities and the principal investigator reminded the staff and co-researchers that they could choose to sit out during the activities or not attend the sessions. However, the majority of the staff and co-researchers continued to attend and participate in the activities as best as they could manage. Additionally, many were confused about the purpose of such sessions, especially since the connection between some of the activities and the production of a forum was not always immediately apparent, despite our discussions on this topic.

In retrospect, the activities allowed us to share laughter, be more vulnerable with each other, and develop stronger bonds as a team. While the committee had been meeting regularly for over a year, there were many things we still did not know about each other that came to light during these sessions. Additionally, newer committee members were able to feel rapidly connected to the group and were noted to speak up and participate quite readily. Our group debriefs and discussions about Forum Theatre confirmed that CDAC members recognized the potential positive impact of hosting a Forum as a way to increase awareness and knowledge of diabetes with the Forum’s audience. The possibility of having a positive impact for some and the excitement of trying something new for others, led the committee to continue to the next steps of preparing to produce the script to be performed in the Forum.

### Script development and rehearsals: tension in getting it right

The committee was generally enthusiastic to enter the next stage of the Forum Theatre process. This was especially true for individuals who had not fully understand the purpose of the drama workshops, as they thought the script creation and rehearsals were a more productive use of time than the preceding workshops. However, tensions rose quickly when first reviewing and editing the script. Committee members shared concerns that the performance would not be representative of their individual lived experiences, including the distinct lived experiences of having type 1 versus type 2 diabetes and their differing experiences of stigma.

While people living with any form of diabetes are prone to hyperglycemia (i.e., high blood glucose), people with type 1 diabetes face unique challenges due to required treatment with insulin therapy. In contrast, many people with type 2 diabetes are not entirely dependent upon insulin injections. As a result, those with type 1 diabetes more commonly face stigma related to insulin injections, symptoms of low blood glucose levels (hypoglycemia), or the need to self-monitor blood glucose levels. Conversely, those with type 2 diabetes more commonly encounter stigma rooted in perceptions of personal responsibility, such as feeling as though they are to blame for their diabetes due to body size or health-related practices.

Some CDAC members with type 2 diabetes expressed difficulty relating to the experiences of people with type 1 diabetes, which lead to moments of tension when some members who had type 1 diabetes began referring to these with type 2 diabetes pejoratively as “fake” or “wanna-be diabetics.” These tensions were resolved through several discussions and reflections, during which the committee agreed that the Forum was not meant to represent one person’s lived experiences but the challenges the group had faced as a collective. Although the specific events around the experiences of stigma differed, all members connected with feelings of worry about their diabetes management and being blamed or dismissed. In recognition of these shared experiences and goals, the team chose not to specify a type of diabetes in our production and to keep it open-ended and focused on experiences that were common among the group, rather than those that would only apply to people with one type of diabetes but not the others.

With consensus among the group, we held multiple rehearsals in the same space where the Forum Theatre performance would take place, we refined elements such as body language, placement on stage, use of props, and the volume of our speech until everyone felt comfortable and ready to perform on stage in front of an audience. Deciding who would play which character depended on the committee members’ comfort levels, as some members shared that they wanted minor speaking roles where they could sit while delivering their lines (due to the use of mobility aids or physical limitations). In contrast, others preferred having prominent speaking roles where they could move around the stage. Co-researchers who preferred not to have speaking roles were given the choice to sit with the audience or participate as a background character in a scene, and research staff filled in roles as needed. As rehearsals progressed, some co-researchers felt increased pressure and excitement to master their performance and memorize their lines to the best of their ability to do justice to the group’s efforts. Others volunteered to come in earlier to set up props and print and highlight scripts. The momentum created by each committee member kept others in the group motivated and enthusiastic to participate in and host the Forum.

### The forum: putting ourselves out there

Across both Forums in 2023, more than 150 people, or spect-actors, attended. Prior to beginning the performance, we held a breakfast for the CDAC members, which fostered a welcoming atmosphere and a sense of ease among the co-researchers as they prepared to perform. This inviting environment was pivotal in mitigating nervousness within the group and helped cultivate an environment of trust and camaraderie. At the beginning of each performance, the stage manager shared with the spect-actors that we would run through the performance in its entirety once without any interruptions, and then on the second run through spect-ators would be able to jump in by yelling “stop” and intervene when they noticed opportunities for change. After each intervention, the scenes continued as scripted, allowing the spect-actors to continue intervening in later scenes. In total, those attending the Forums generated 20 unique interventions by interrupting the scripted Forum and suggesting alternative actions.

Among the interventions, 12 consisted of spect-actors stepping in as shelter staff and supporting their colleagues as they took breaks or suggesting possible ways to address their colleagues’ assumptions about the clients. Another six spect-actors intervened by portraying the role of other clients in the shelter setting, showcasing the significance of peer advocacy. Lastly, two interventions drew attention to the limited access to medications within shelters, particularly focusing on the insufficient access to insulin due to safe storage in emergency housing shelters. These spect-actors questioned policies and put forth compelling solutions that could enable individuals to have improved access to vital supplies within the shelter setting. Overall, the interventions from both Forums can be split into two groups: resource-based and social. The resource-based interventions touched upon the (limited) availability and funding for staff, nutritious food, and juice boxes (for low blood sugar emergencies). Social interventions focused on fostering staff awareness and education, as well as empowering clients and bystanders to advocate for those being shamed, judged, or experiencing low or high blood sugars. Interestingly, some spect-actors, alongside the stage director, considered the resource-based interventions to be “magical” interventions (21) characterized by the sudden and unexplained appearance of available staff, donation of nutritious food, or a conveniently placed juice box in the scene. While such “magical” interventions are often discouraged in Forum Theatre (21), members of the CDAC noted that these moments still held value, and highlighted the need for upstream, systemic change, particularly policy reform to address the root causes of resource scarcity.

Overall, the Forums were lively and filled with active participation and dialogue between the co-researchers and the spect-actors. These interactions created multiple moments of teaching, led by the co-researchers, and demonstrated the strength of Forum Theatre in centering the co-researchers’ collective experiences with diabetes stigma, elevating their voice and expertise. The co-researchers felt in charge of the Forum and were able to share their lived experiences in a way that felt empowering rather than dehumanizing. Additionally, the co-researchers used their discretion in their speaking tone and added non-scripted lines, especially during interventions, to entice reactions of laughter, empathy, and concern from the audience. The informal feedback we received from the spect-actors was positive and supportive, with many appreciating the co-researchers’ strength in sharing their most challenging experiences, the portrayals of which resonated deeply with the spect-actors/audience members.

## Discussion

Through our experiences of utilizing Forum Theatre to raise awareness of the stigma experienced by individuals with diabetes who are experiencing homelessness, we found our process to consist of three distinct phases with unique reflections, challenges, and successes, including (i) Drama workshops: Willingness to try something new; (ii) Script development and rehearsals: Tension in getting it right; and (iii) The Forum: Putting ourselves out there. Overall, our experiences were similar to those described in previous research demonstrating Forum Theatre’s effectiveness in raising awareness, reducing stigma, and promoting practical solutions (6–8, 22). However, we highlighted key instances of group friction and ways to move forward during the Forum Theatre process, which may be helpful to future researchers considering its use.

Issues that arose during our Forum Theatre process were expected as some level of tension between group members can inevitably arise during community-based group projects. One noteworthy consideration for researchers engaging a large group of people with lived experience is recognizing the diversity of perspectives and experiences. In our case, the co-researchers had different levels of enthusiasm at each stage and had different experiences with their diabetes that they wanted to showcase in the performance. We found that resolving these differences and moving forward with the project was possible by facilitating an equal and fair experience for all those involved. Using a living ‘Terms of Reference’ document inspired by the tenets of CBPR. Having this document, which each committee member agreed to at the beginning of the project was helpful in maintaining a respectful and productive team atmosphere (18). This document outlined rules or agreements that were important to all committee members, including respecting everyone’s identity and voice, recognizing collective ownership of the project, scheduling regular debriefs, having the flexibility to adapt participation, and having their time spent in meetings and rehearsals be recognized with an hourly honorarium. We consistently revisited this document as a group and implemented the points in it as best we could. While the Terms of Reference provided structure and guidance, it is important to note that the Forum Theatre process inherently built team capacity and communication skills through the drama workshops. Through the collaborative nature of the drama workshops, committee members engaged in active problem-solving, negotiation, and role-play, which fostered a deeper understanding and awareness of each other’s strengths, weaknesses, and values, contributing to the team’s overall cohesiveness.

Lastly it is important to acknowledge that despite the successes and positive reception of our Forum Theatre productions, the financial, time, and human resources needed to stage a single performance made it unlikely that we could host serial or ongoing productions. Recognizing these resource constraints, the committee explored alternative arts-based methods that could offer a more sustainable and wider-reaching platform for the co-researchers’ stories. The committee quickly landed on using film, as it was seen to allow us to preserve the key moments from the live Forum Theatre performance while extending its reach beyond the immediate audience who may not have been able to attend the performance in person. This shift to film allowed the project to continue influencing discussions about stigma, healthcare access, and the intersections of homelessness and chronic illness long after the performances themselves, and in a wider variety of places than could have been reached with Forum Theatre alone.

## Conclusion

Overall, using Forum Theatre as a CBPR group allowed us to generate valuable insights into the stigma surrounding homelessness and diabetes as well as possible solutions through resource-based or social interventions. The process of developing and hosting the Forum provided a practical example of the strengths and challenges of arts-based methods. In our case, this included exploring our willingness to try something new, navigating tensions, telling a representative story, and going outside of our comfort zones. Future researchers considering using Forum Theatre are encouraged to consider these experiences when planning and navigating their own productions.

## Data Availability

Data are not available due to local ethics requirements. Requests to access the datasets should be directed to David Campbell, dcampbel@ucalgary.ca.
